# OCT Angiography and Ellipsoid Zone Mapping of Macular Telangiectasia Type 2 From the AVATAR Study

**DOI:** 10.1167/iovs.16-20976

**Published:** 2017-07

**Authors:** Anne P. Runkle, Peter K. Kaiser, Sunil K. Srivastava, Andrew P. Schachat, Jamie L. Reese, Justis P. Ehlers

**Affiliations:** 1 Ophthalmic Imaging Center, Cleveland Clinic, Cleveland, Ohio, United States; 2 Cole Eye Institute, Cleveland Clinic, Cleveland, Ohio, United States

**Keywords:** OCT-A, macular telangiectasia, JXT, ellipsoid zone, OCT

## Abstract

**Purpose:**

To evaluate alterations on optical coherence tomography angiography (OCT-A) and quantitatively assess alterations in the ellipsoid zone (EZ) in eyes with macular telangiectasia type 2 (MacTel type 2).

**Methods:**

The Observational Assessment of Visualizing and Analyzing Vessels With Optical Coherence Tomography Angiography in Retinal Diseases study is an institutional review board-approved prospective, observational study investigating OCT-A in macular disease. Patients underwent spectral-domain (SD)-OCT and OCT-A imaging at a single visit. SD-OCT data were analyzed using a novel OCT EZ-mapping software to obtain linear, area, and volumetric measurements of the EZ-retinal pigment epithelium (RPE) complex across the macular cube. OCT-A retinal capillary density was measured using the Optovue Avanti split-spectrum amplitude-decorrelation angiography algorithm. EZ-RPE parameters were compared to age-matched, sex-matched controls.

**Results:**

Fourteen eyes of seven patients (mean age, 59 ± 6.5 years) were analyzed. Mean visual acuity was 20/45 (range, 20/20–20/150). EZ-RPE central foveal mean thickness was 27.8 ± 6.7 μm, EZ-RPE central foveal thickness was 22.1 ± 21.6 μm, EZ-RPE central foveal area was 0.17 ± 0.04 mm^2^, and EZ-RPE central subfield volume was 0.017 ± 0.012 mm^3^. Each of these measurements was significantly inversely correlated with visual acuity (*P* < 0.02). In addition, all of these measurements were significantly reduced compared to controls (all *P* ≤ 0.005). OCT-A showed a reduced parafoveal vessel density of 50.8% temporally compared to 53.8% nasally (*P* = 0.01) in the superficial vascular plexus. In the deep vascular plexus, similar findings were noted with a parafoveal vessel density of 56.7% temporally and 58.8% nasally (*P* = 0.01).

**Conclusions:**

Abnormalities in EZ-RPE thickness, area, and volume are correlated with visual acuity in MacTel type 2, and may provide quantitative markers to measure disease progression and treatment response. OCT-A was a useful adjunct for determining disease severity.

Macular telangiectasia type 2 (MacTel type 2) is a bilateral retinal disease that primarily affects juxtafoveal region of the macula. It is usually diagnosed in the fifth or sixth decade of life, and patients typically present with symptoms of scotoma, metamorphopsia, and decreased vision.^1^ It is traditionally diagnosed and staged according to the Gass classification system using fluorescein angiography, which may show juxtafoveal ectatic capillaries (often in the temporal region) in the early phase and diffuse hyperfluorescence in the late phase.^2^

In recent years, optical coherence tomography (OCT) and OCT angiography have been examined as potential imaging modalities to facilitate the diagnosis and characterization of MacTel type 2.^3–18^ Spectral-domain (SD)-OCT demonstrates characteristic features of MacTel type 2, including hyporeflective cavities and atrophy of the neurosensory retina, ellipsoid zone (EZ) breakdown, increased reflectivity of inner retinal layers, and retinal thinning.^1^ Of particular interest is the EZ, which has been shown to be disrupted in MacTel type 2 and may serve as a marker to measure disease progression and treatment response.^10,11,13,14,19^ Quantitative assessment of the EZ features has been historically limited due to challenges in outer retinal segmentation. Recent technology development in EZ mapping software has allowed new opportunities in quantitative EZ assessment.^20^

OCT angiography (OCT-A) has also been used to examine MacTel type 2. OCT-A is a novel, noninvasive imaging method that analyzes high-speed OCT images to measure changing reflectance and reconstruct high-resolution blood flow maps of the retina, allowing en face visualization of the retinal capillary network. Studies investigating the features of MacTel type 2 using OCT-A have demonstrated alterations in the superficial and deep retinal capillary plexuses, including capillary dilation, abnormal anastomoses, telangiectasias, and decreased capillary density.^3–7,12,18^

In this study, EZ mapping and the OCT-A findings in MacTel type 2 are described and correlated.

## Methods

### Imaging Protocol

The Observational Assessment of Visualizing and Analyzing Vessels With Optical Coherence Tomography Angiography in Retinal Diseases (AVATAR) study is an institutional review board-approved prospective, observational study investigating the use of OCT angiography in patients with macular disease. Patients were recruited adult patients undergoing OCT testing for standard-of-care management. Written informed consent was obtained from all participants. For this analysis, all patients in the AVATAR study with a diagnosis of MacTel type 2 were identified. Diagnosis of MacTel type 2 was based on clinical features, SD-OCT findings, and fluorescein angiography.

**Figure 1:**
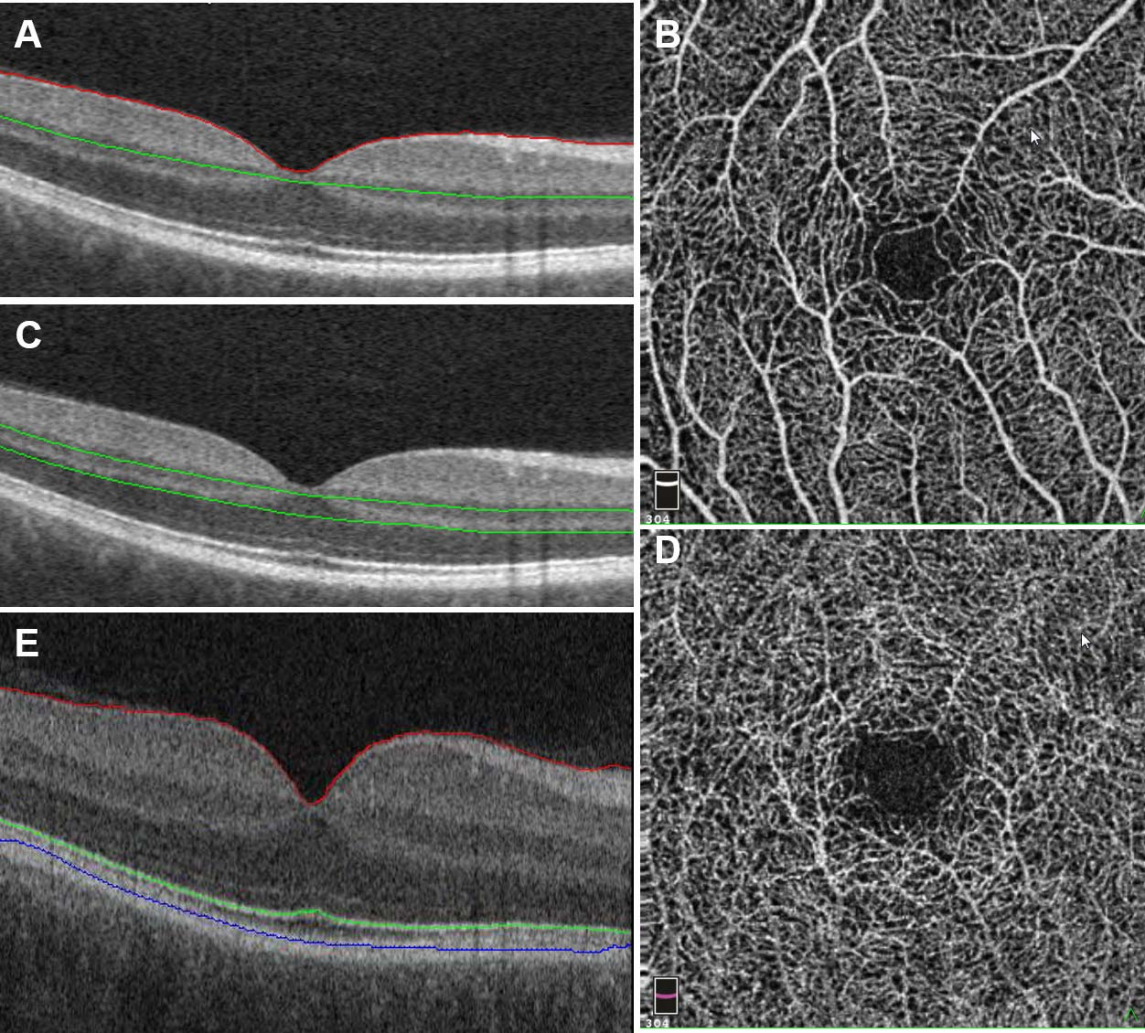
Normal scan and segmentation example. (**A**) Central foveal B-scan of a control eye, showing the OCT-A segmentation for the superficial capillary plexus. (**B**) Corresponding superficial capillary plexus OCT-A. (**C**) Central foveal B-scan of a control eye, showing the OCT-A segmentation for the deep capillary plexus. (**D**) Corresponding deep capillary plexus OCT-A. (**E**) Central foveal B-scan of a control eye, showing EZ-mapping segmentation including nerve fiber layer (*red*), EZ (*green*), and RPE (*blue*).

All patients underwent imaging of both eyes with the Avanti RTVue XR HD (Optovue, Fremont, CA, USA) and the Cirrus 5000 SD-OCT system (Carl Zeiss Meditec, Dublin, CA, USA). For the AVATAR protocol, an OCT-A cube with 3.00-mm scan lengths was obtained. The Avanti system was equipped with prototype split-spectrum amplitude-decorrelation angiography (SSADA) algorithm software to generate segmented retinal OCT-A scans with associated perfusion density mapping software. The system provides en face visualizations of the superficial and deep capillary layers, as well as the outer retina and the choroid vasculature (Fig. 1). The quantitative measurements of interest from the algorithm included vessel density of the superficial and deep inner retinal vascular plexuses, which was further categorized into temporal, nasal, superior, and inferior regions. The reports generated by the OCT images for retinal layer analysis were obtained with the Cirrus 5000 SD-OCT system using the macular cube 512 × 128 scan and HD five-line raster horizontal and vertical patterns.

### Image Analysis

OCT-A reports and volumetric review of OCT-A data sets were performed for analysis of retinal vascular abnormality. Retinal vascular perfusion density analysis was performed using the proprietary AngioAnalytics (Optovue) vessel density algorithm. Area of outer retina capillary invasion on 3 mm × 3 mm OCT-A scans was measured in ImageJ.

Cirrus HD-OCT images were exported into proprietary OCT analysis software platform for EZ mapping.^20^ Image processing and analysis generated quantitative values of the following parameters of interest: EZ-retinal pigment epithelium (RPE) central foveal thickness and mean thickness, EZ-RPE central foveal area, and EZ-RPE central subfield volume, as previously described (Fig. 1).^20^ EZ-RPE thickness was defined as the height (mm) from the middle of the RPE boundary to the middle of the EZ. EZ-RPE central foveal area was defined as the total area (mm^2^) between the middle of the RPE boundary and the middle of the EZ on the central foveal slice. EZ-RPE central subfield volume was defined as the total volume (mm^3^) between the middle of the RPE boundary and the middle of the EZ in the central subfield. EZ attenuation and atrophy were defined as the percentage of the total area where the EZ-RPE thickness was less than 20 μm or equal to 0 μm, respectively. Volumetric assessment of total retinal fluid (e.g., intraretinal fluid and subretinal fluid) was also performed in each SD-OCT scan.^21^ These same EZ measurements were also obtained for 14 age- and sex-matched control eyes from a previously collected normative SD-OCT database (Dukles NA, et al. *IOVS* 2017:ARVO E-Abstract 1887).

### Statistical Analysis

All statistical calculations were performed using JMP Pro 12.1.0 Software (SAS Institute, Inc., Cary, NC, USA) or Microsoft Excel (Microsoft, Redmond, WA, USA). Regional differences in OCT-A vessel density were compared using the Wilcoxon signed rank test. Age- and sex-matched comparison of EZ measurements was performed using the Wilcoxon signed rank test. Correlation between visual acuity (VA) and EZ-RPE architectural measurements was measured using the Spearman rank correlation coefficient. Correlation between area of outer retinal vessel proliferation and EZ attenuation and atrophy was also measured using the Spearman rank correlation coefficient.

## Results

### Demographics and Clinical Characteristics

Fourteen eyes of seven patients diagnosed with MacTel type 2 were identified in the AVATAR study (Table 1). The mean patient age was 59 years (range of 52–69). The mean visual acuity was 20/45 with a range of 20/20 to 20/150, with seven eyes (50%) having a visual acuity of 20/40 or better. No patients had previously diagnosed choroidal neovascularization.

**Table 1:**
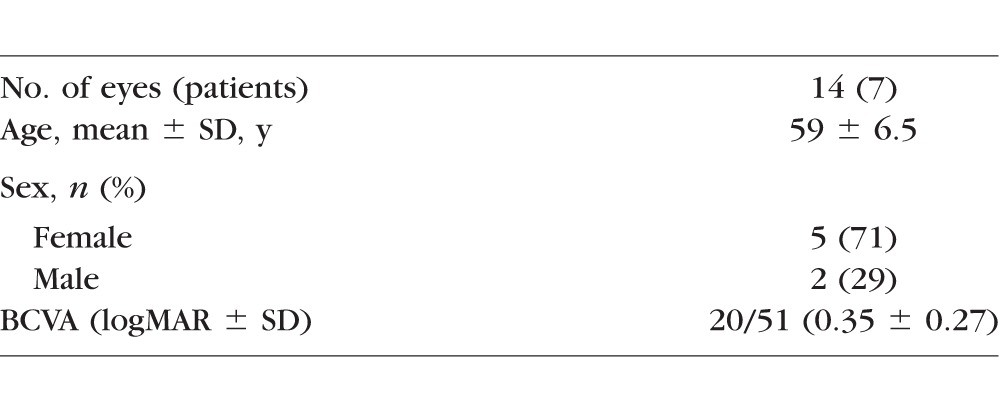
Clinical and Demographic Data of Patients With MacTel Type 2 in the AVATAR Study

### OCT-Angiography Analysis and Functional Correlation

**Figure 2:**
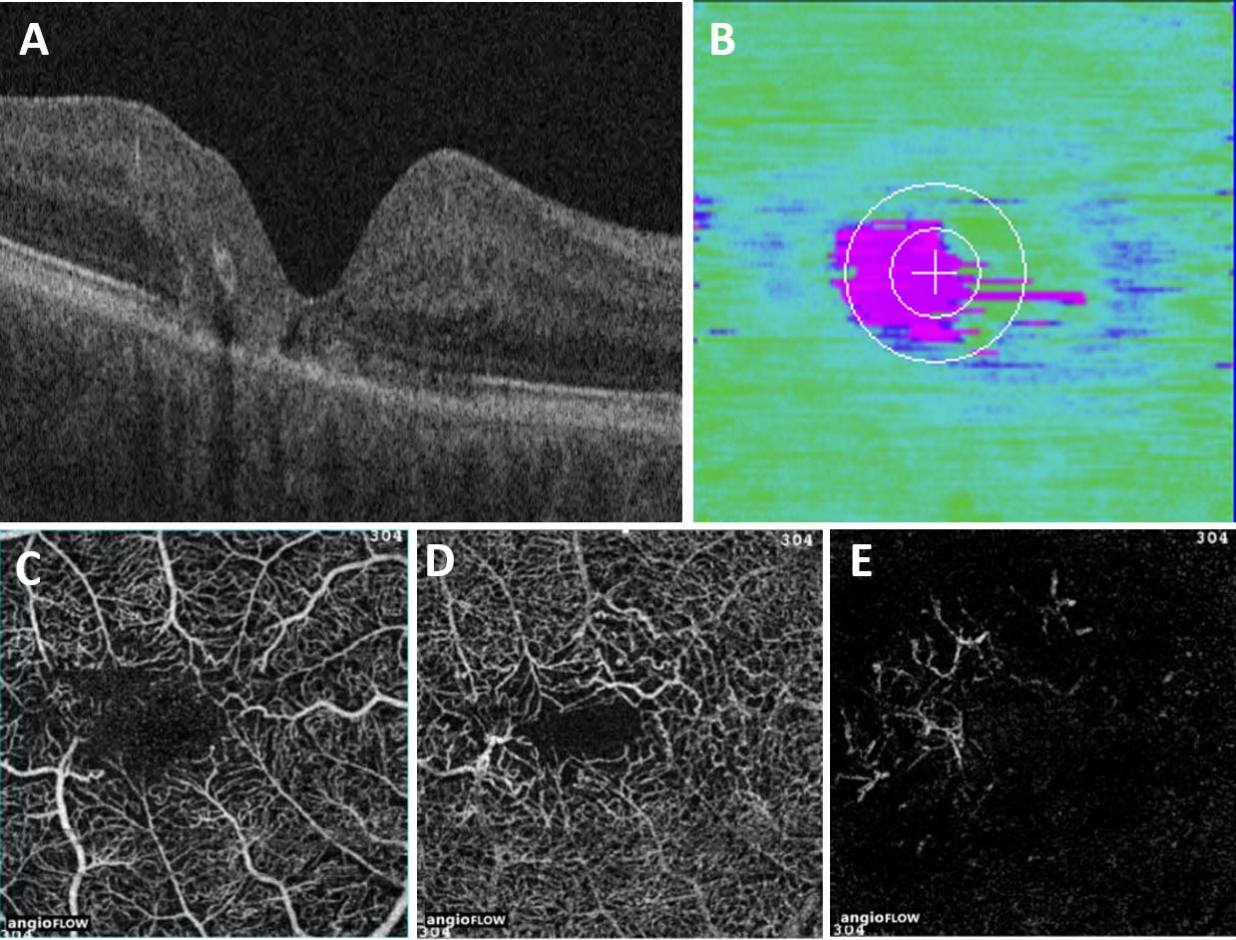
Advanced MacTel type 2 with EZ mapping and OCT-angiography. (**A**) Central foveal B-scan of an eye with MacTel type 2, showing EZ disruption. (**B**) EZ- RPE thickness map of the same eye, showing central foveal and temporal EZ atrophy (i.e., areas of pink). OCT-A of the same eye shows right angle vessels and temporal flow voids in the superficial capillary plexus (**C**), increased vascular diameter and tortuosity in the deep capillary plexus (**D**), and (**E**) capillary invasion of the outer retina. Note the corresponding size and location of the EZ atrophy and the vascular abnormalities.

**Figure 3:**
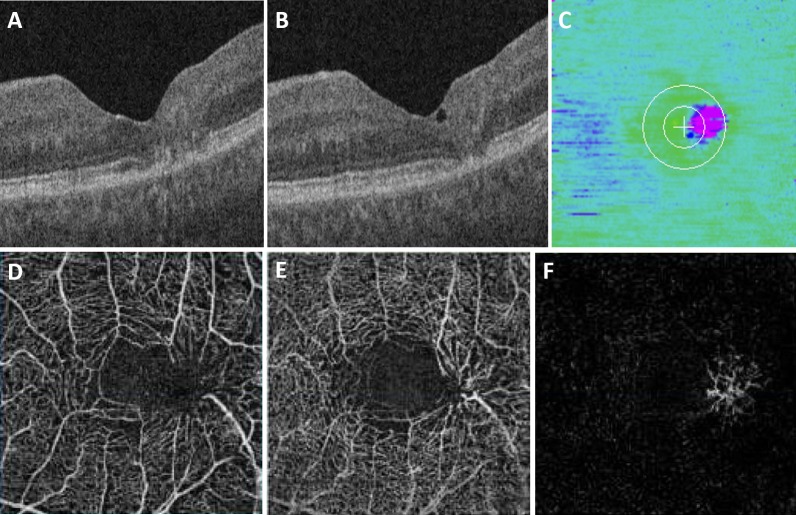
Moderate MacTel type 2 with EZ mapping and OCT-angiography. (**A**) Central foveal B-scan of an eye with MacTel type 2, showing disruption of the EZ. (**B**) B-scan of the same eye more superiorly, showing focal EZ disruption and a small area of intraretinal fluid. (**C**) EZ-RPE thickness map of the same eye, showing juxtafoveal temporal EZ atrophy (*pink*). OCT-A of the same eye shows right angle vessels and increased vascular caliber and increased flow voids in both the superficial (**D**) and deep (**E**) capillary plexuses. The outer retina segmentation identifies flow within a temporal region of capillary invasion (**F**). Note the corresponding size and location of the EZ atrophy and the vascular abnormalities.

**Figure 4:**
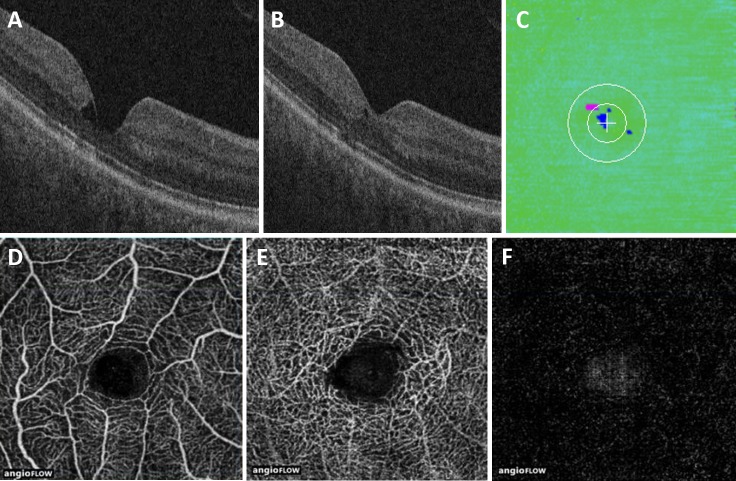
Mild MacTel type 2 with EZ mapping and OCT-angiography. (**A**) Central foveal B-scan of an eye with MacTel type 2, showing a large hyporeflective cavity and an intact EZ. (**B**) B-scan of the same eye more superiorly, showing EZ disruption and a smaller hyporeflective cavity. (**C**) EZ-RPE thickness map demonstrates intraretinal fluid (*blue*), but minimal EZ atrophy (*pink*). OCT-A of the same eye shows minimal changes within the superficial capillary plexus (**D**). The deep capillary plexus shows enlargement of the vasculature with possible microaneurysms (**E**). The outer retina does not exhibit any abnormal flow patterns (**F**).

OCT-A scans were noted to have abnormalities with a predilection to the temporal juxtafoveal area. These findings included vessel dilation, capillary irregularity, and variable outer retinal vascularization (Figs. 2–4). In both the superficial and deep vascular plexuses, the parafoveal temporal vessel density was found to be less than the parafoveal nasal vessel density (Table 2; Figs. 2, 3). In the superficial vascular plexus, the parafoveal vessel density was 50.8% in the temporal region and 53.8% in the nasal region (*P* = 0.01). In the deep vascular plexus, the parafoveal vessel density was 56.7% in the temporal region and 58.8% in the nasal region (*P* = 0.01). Vascular density did not correlate with visual acuity.

**Table 2:**
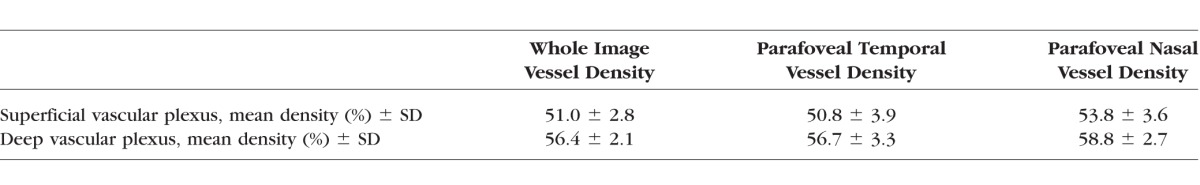
Vessel Density in the Superior and Deep Inner Retinal Vascular Plexuses

### EZ-Mapping and Functional/Structural Correlation

Nine (64%) of the 14 eyes had inner retinal cysts and six (43%) had outer retinal cavitation. EZ-RPE central foveal thickness was 22.1 μm ± 21.6 μm, EZ-RPE central foveal mean thickness was 27.8 μm ± 6.7 μm, EZ-RPE central foveal area was 0.17 mm^2^ ± 0.04 mm^2^, and EZ-RPE central subfield volume was 0.017 mm^3^ ± 0.012 mm^3^.

Age- and sex-matched comparison between eyes with MacTel type 2 and normative controls showed that eyes with MacTel type 2 had significantly decreased EZ-RPE central foveal thickness, central foveal mean thickness, central foveal area, and central subfield volume, as well as significantly increased area of EZ attenuation and atrophy (Table 3).

**Table 3:**
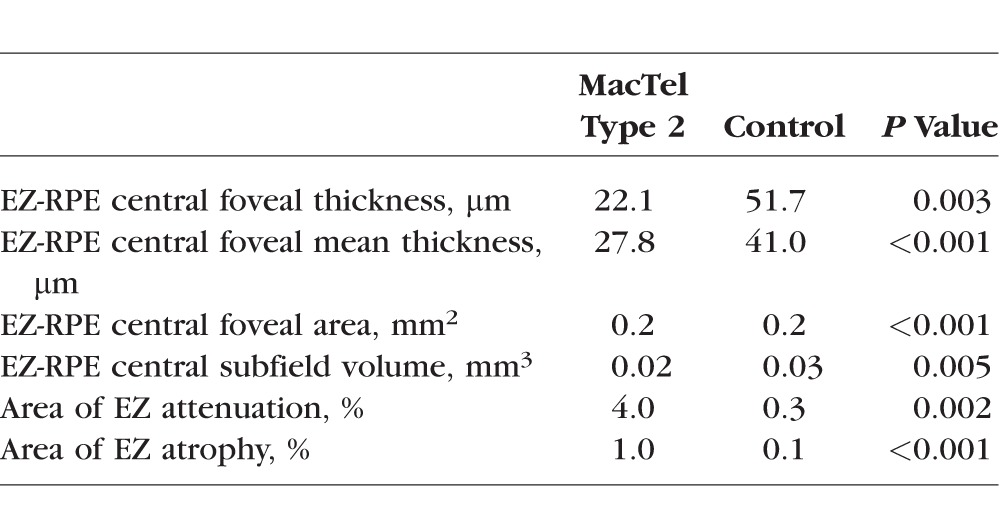
Age- and Sex-Matched Comparison of Outer Retinal Measurements Between MacTel Type 2 and Normative Control Eyes

Correlation of EZ-RPE parameters with visual acuity determined that each EZ-RPE feature was significantly inversely correlated with VA (Table 4). Retinal fluid volume was not associated with visual acuity (*P* = 0.07), but trended toward significance.

**Table 4:**
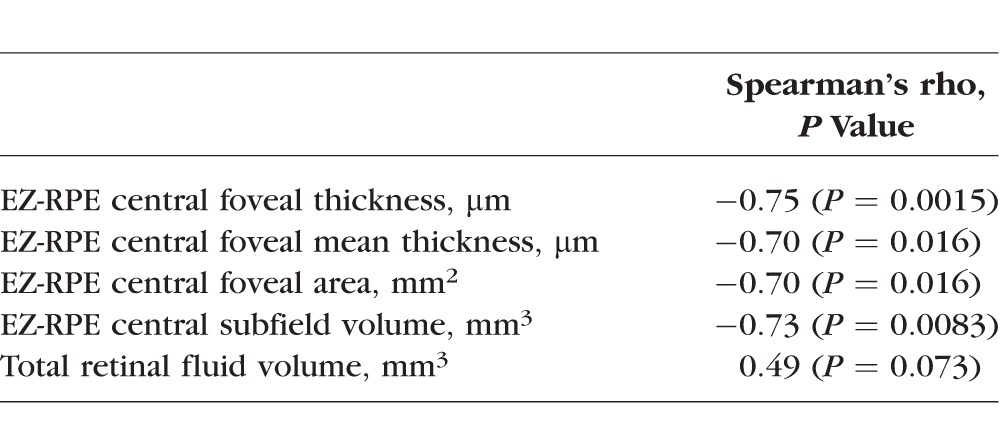
Spearman Rank Correlation Coefficient With Visual Acuity for EZ-RPE Measurements

Correlation of EZ atrophy with area of outer retina capillary proliferation was found to be 0.765 (*P* = 0.002) and correlation of EZ attenuation with area of outer retina vasculature proliferation was found to be 0.685 (*P* = 0.007).

## Discussion

Recent advances in SD-OCT and OCT angiography technologies have revolutionized the diagnosis and management of ophthalmic disease. In addition, the data obtained from these scans provide quantitative measurements of retinal layer alterations, allowing us to further understand the pathophysiology of disease processes. Of particular interest for MacTel type 2 is SD-OCT measurement of the EZ and outer retina, previously referred to as the inner segment/outer segment junction. The integrity of the EZ has been correlated with visual acuity and prognosis across a wide variety of diseases, from multiple evanescent white dot syndrome to age-related macular degeneration to hydroxychloroquine retinopathy.^20,22–25^

Previous reports on MacTel type 2 have found that EZ disruption is correlated with decrease in cone density, and that that loss of EZ integrity is associated with decreased retinal sensitivity.^10,11,13,14,16–18,26^ While the pathophysiology of MacTel type 2 remains uncertain, it is hypothesized to be linked to Müller cell death or dysfunction, which leads to the resulting photoreceptor loss (and thus EZ disruption) and juxtafoveal capillary proliferation.^1,18^

While many studies have examined EZ disruption as a marker of MacTel type 2 severity, quantification of the EZ disruption has not been described and is not currently widely available. Several studies used a binary measure of whether the EZ is intact or disrupted at discrete points on the macula, and two studies correlated this binary measure with retinal sensitivity on microperimetry.^14–17,23^ Other studies have measured the en face total area of the EZ disruption, or have reported on the co-localization of area of EZ breakdown and proliferation of abnormal capillary vessels.^10,11,13,18^ Our study adds to the current knowledge of EZ disruption in MacTel type 2 by investigating EZ mapping technology for quantifying EZ breakdown.^20^ First, we found that EZ-RPE thickness, area, and volume measurements in eyes with MacTel type 2 were significantly decreased when compared to age- and sex-matched normative controls, and percentage of EZ atrophy and attenuation were significantly increased when compared to normative controls (Table 3). We also found that EZ-RPE central foveal thickness, EZ-RPE central foveal mean thickness, EZ-RPE central foveal area, and EZ-RPE central subfield volume were all significantly correlated with visual acuity (Table 4). The strongest predictors of visual acuity were EZ-RPE central subfield volume and EZ-RPE central foveal thickness. From a pathophysiology standpoint, these are logical measures for photoreceptor loss: patients with the poorest visual acuity tended to have complete EZ loss in the central subfield (Fig. 2). In addition, we found that there was a strong correlation between the area of outer retina capillary proliferation and the area of EZ loss. This builds on previous findings described qualitatively by Gaudric et al.,^18^ which noted that “deep retinal capillaries invaded the outer retina specifically in the area of EZ loss.”

In this report, OCT-A was valuable for providing both quantitative and qualitative data for evaluating patients with MacTel type 2. In both the superficial and deep retinal capillary plexuses, capillary density was significantly decreased temporally, which is consistent with underlying disease features. However, this capillary density change was not associated with visual acuity. One other study by Chidambara et al.^27^ has also quantified vessel density in MacTel type 2 using a MATLAB-based analysis rather than the Avanti SSADA algorithm. Their report found lower vessel density than found in our study (39.99% and 39.03% in the superficial and deep plexuses, respectively), but this difference is may be attributable to differences in disease severity and/or algorithmic calculation differences.

From a qualitative perspective, OCT-A findings in our study confirmed what has been previously reported, including vessel dilation, capillary thinning, and telangiectasias.^1,3–5,12^ OCT-A and SD-OCT in combination was especially useful in evaluating disease severity. In advanced MacTel type 2 (Fig. 2), SD-OCT and OCT-A findings provide complementary information: SD-OCT shows central EZ loss (and thus decrease in EZ-RPE thickness, area, and volume), while OCT-A demonstrates flow abnormalities with telangiectasias and flow voids in the capillary plexuses and capillary invasion in the outer retina. In less advanced MacTel type 2 (Fig. 3), SD-OCT demonstrates disruption of the EZ centrally, although the central foveal EZ-RPE thickness is normal, because the area of EZ loss is slightly temporal. The OCT-A images augment the SD-OCT images by demonstrating flow defects and telangiectatic vessels with alterations in vessel caliber and tortuosity in both superficial and deep retinal capillary plexuses, and vascular invasion in the outer retina. Mild-moderate MacTel type 2 exhibited very minimal, patchy EZ loss on SD-OCT, but retinal vascular alterations in tortuosity and flow abnormalities were clearly present in the temporal retina on OCT-A (Fig. 4). Minimal vascular flow was identified in the outer retina. While many studies examine the use of SD-OCT or OCT-A for evaluation of MacTel type 2, our study suggests that there may be benefit to using the two in conjunction.

There are important limitations to this study. The sample size of patients enrolled with MacTel type 2 is small. In addition, the image data we present here represents a cross-sectional view of the disease: one patient visit where they received both SD-OCT and OCT-A imaging. Therefore, it was not possible to determine how parameters such as EZ-RPE measurements or capillary density change over the course of their disease. The use of Snellen VA is not the ideal parameter, as functional testing such as microperimetry or standardized protocol refraction visual acuity may provide a more sensitive measurement of retinal function in MacTel type 2.^16,28^

Despite these limitations, we believe that this study provides a novel contribution toward quantitative measurements for the diagnosis and monitoring of MacTel type 2. SD-OCT and OCT-A provide new methods of noninvasive imaging of the perifoveal area in this disease, and may provide techniques for disease staging and monitoring of therapeutic response. SD-OCT EZ-RPE measurement abnormalities, including central foveal thickness, central foveal area, and central subfield volume, are significantly correlated with visual acuity in MacTel type 2. In addition, OCT-A demonstrates the ability to quantify capillary density and to provide noninvasive vessel images to augment SD-OCT in understanding disease progression.
